# Dietary Deoxynivalenol Exposure Assessment in University Students from Japan

**DOI:** 10.14252/foodsafetyfscj.2018021

**Published:** 2019-06-28

**Authors:** Lei Xia, Yoshiko Sugita-Konishi, Yunyun Gong, Michael Routledge

**Affiliations:** 1 School of Food Science & Nutrition, University of Leeds, Leeds, UK; 2 Department of Food and Life Sciences, Azabu University, Japan; 3 School of Medicine, University of Leeds, Leeds, UK

**Keywords:** biomarker, deoxynivalenol, exposure, mycotoxin

## Abstract

This study was conducted to give a preliminary estimation of deoxynivalenol (DON) dietary exposure in Japanese university students (n = 30, aged 22–25 years) using a biomarker approach and to examine the correlation between wheat food intake and DON exposure levels. Spot urine samples were collected from 30 students of Azabu University, Tokyo. Urine samples were treated with enzyme digestion (for total DON measurement) and without (for unconjugated DON analysis) before clean-up using an immuno-affinity column and analysis using an LC-MS method, with a ^13^C_15_- DON internal standard used for accurate quantification. The limit of detection for this method is 0.5 ng/mL urine. The geometric mean (95% CI) of DON concentration was 2.03 (1.64 – 6.87) ng per mL urine. Ninety of the urine samples had detectable levels of urinary DON. The DON dietary intake exposure estimation suggested that one out of the 30 subjects had an intake of DON that exceeded Joint FAO/WHO Expert Committee on Food Additives (JECFA) provisional maximum tolerable daily intake (PMTDI) level. Mean ratio of free DON to total DON was determined to be 19%. Wheat intake assessed using a basic food frequent questionnaire method did not show a significant correlation with the urinary DON level.

## Introduction

Deoxynivalenol (DON), produced by *Fusarium graminearum* and *Fusarium culmorum,* is a common type B trichothecene mycotoxin which frequently contaminates wheat, barley, oats, maize and other grains, particularly in the global north temperate environment. Recently the Food Safety Commission of Japan (FSCJ) reported the estimated exposure in Japan is likely to be close to the provisional maximum tolerable daily intake (PMTDI) (1 μg/kg bw per day for DON) set by the Joint FAO/WHO Expert Committee on Food Additives (JECFA), particularly in children, and highlighted the need for more human exposure and epidemiology data^1^^)^. The major metabolites of DON in humans include DON-3-glucuronide (DON-3-GlcA), DON-15-glucuronide (DON-15-GlcA) and C12,13-deepoxy deoxynivalenol (DOM-1) and are mainly excreted via the faeces and urine^2^^,^^3^^)^. Due to the high and rapid excretion rate of DON through urine, urinary DON is the major biomarker for assessing DON exposure^4^^,^^5^^,^^6^^)^. The conjugated form of DON is the predominant form of DON metabolites in urine and only about 25% of the DON metabolites exist in the urine as its free form. Enzyme hydrolysis during the extraction step has been proposed in order to increase the accuracy in determining DON levels in urine, which converts the conjugated DON back to its free form so that total urinary DON is measured to assess the exposure^7^^)^. Total DON (extraction with enzyme digestion), free DON, DON-3-GlcA and DON-15-GlcA are all suitable biomarkers for DON exposure assessment, while DOM-1 showed a very low concentration in human urine samples^8^^,^^9^^,^^10^^,^^11^^,^^12^^,^^13^^,^^14^^,^^15^^)^. The benefit of utilizing biomarker measurement, compared to the traditional dietary exposure assessment, to assess the DON exposure is that this method can cover DON exposure through different exposure routes.

## Materials and Methods

### Study Population and Sample Collection

The present work is a pilot study involving university students from Tokyo, Japan who were potentially exposed to DON in their normal diet. The 30 university student participants provided their consent to be interviewed and physically examined. Ethical approval was obtained from Azabu University where the urine samples were collected. The spot urine samples were collected from all participants in July 2017, in a labeled sterile 50 mL polyethylene urine collection container and kept in an ice box. All urine samples were then immediately transferred to be stored at the Azuba University Laboratory at −20°C. All the samples were separated to two 20 mL portions, freeze-dried and then shipped with dry ice to University of Leeds, UK, by air and stored at −20°C. Among the 30 participants, 18 of them are males and 12 of them are females, all aged between 22 and 25 years of age. The students were given a very brief dietary recall questionnaire. The questionnaire simply asked if the student had breakfast/lunch/afternoon tea/dinner on the day before urine sample collection or not and how many wheat products they had consumed for each of those meals. The study was approved by the University of Leeds Mathematics and Physical Sciences and Engineering joint Faculty Research Ethics Committee (MEEC FREC) under the reference number MEEC 17-035 and Azabu University Ethics Committee under the reference number 098.

### Chemicals and Materials

Reference material deoxynivalenol (≥ 98%) and ^13^C_15_ - deoxynivalenol (25 µg/mL in acetonitrile, analytical standard) was purchased from Sigma-Aldrich (Poole, UK). β-glucuronidase (from *Escherichia coli*) was also purchased from Sigma-Aldrich (Poole, UK) for enzymatic hydrolysis of the urine sample. Ammonium formate (97%) was obtained from Acros Organics (Geel, Belgium) and formic acid (≥ 98%) was purchased from Sigma-Aldrich (Poole, UK); these were used for pH modification of the liquid chromatography (LC) mobile phase. Methanol (HPLC grade) was provided by Fisher Scientific (Loughborough, UK) and HPLC grade water was obtained using a Millipore Direct-Q^TM^ water system (Watford, UK).

### Sample Preparation

Freeze-dried urine samples were firstly reconstituted with 20 mL of HPLC grade water leading to x2 more concentrated urine samples. The extraction method for total urinary DON analysis was described in detail elsewhere^16^^)^. The reconstituted urine samples or spiked quality controls (QCs) are centrifuged at 4696 g for 15 minutes at 4°C to remove the impurities. Two mL of the centrifuged samples were aliquoted and the pH of the aliquots were adjusted to pH 6.8. Each urine sample was spiked with 8 ng/mL of ^13^C_15_-deoxynivalenol as the internal standard (IS). Then 1 mL of the spiked urine samples were digested for 18 hours at 37°C with gently mixing, using 5750 units of β- glucuronidase. The enzyme was removed by centrifugation at 4696 g for 15 minutes at 4°C. Digested samples were diluted to 4 mL with phosphate-buffered saline (PBS), pH 7.2, and DON was isolated using DONtest WB immunoaffinity columns (Vicam, Watertown, MA, USA) as per manufacturer’s instructions. DON was eluted from columns with 4 mL methanol, dried *in vacuo*, and reconstituted in 250 µL of 10% ethanol for analysis.

### Standard Solutions and Quality Controls

A stock solution of DON (1 µg/mL) was prepared in 10% (v/v) ethanol. This stock solution was further diluted using 10% ethanol to make the standard solutions with concentrations of 0, 2, 5, 10, 20, 50, 100 and 250 ng/mL. Each of the standard solutions was spiked with 8 ng/mL of ^13^C_15_-DON as IS. Similarly, spiked blank urine sample with DON concentrations of 4, 40, 160 ng/mL were used as the low/medium/high concentration QC samples (also spiked with 8 ng/mL of IS). All the above solutions were stored at −20°C and were brought to room temperature (ca.20°C) before use.

Unknown samples were analyzed in 3 batches of 10 samples with three QC samples per batch (low/medium/high). Four randomly selected samples from the 30 urine samples were re-extracted and analyzed for total DON content, and a coefficient of variation (CV) of the original and repeat data for each re-run sample obtained. The repeated data were in good agreement with the data obtained in the first extraction (mean CV, 4.77%; 95% CI, 3.0–6.6).

### HPLC-MS Conditions

LC was performed on a LC-30AD HPLC separation system (Shimadzu, Milton Keynes, UK) with a Gemini C18 column (110 Å, 4.6 x 250 mm, 5 µm particle size). The column was operated at 30°C. The separation was achieved by elution with an isocratic flow (0.5 mL/min, water: methanol 1:1 (v/v) containing 0.1% formic acid (v/v) and 0.5 mM ammonium formate; 10 minutes). For urine sample analysis, each run also included a wash (water: methanol 1:3 (v/v) containing 0.1% formic acid (v/v) and 0.5 mM ammonium formate; 6 minutes) and a re-equilibration (water: methanol 1:1 (v/v) containing 0.1% formic acid (v/v) and 0.5 mM ammonium formate; 11 minutes). The sample injection volume was 30 µL using an auto-sampler system maintained at ambient temperature.

A LCMS-2020 single quadrupole (Shimadzu, Milton Keynes, UK) was used for mass spectrometry detection. The conditions for the positive electrospray were optimized as follows: Cone gas (nitrogen) 1.5 L/h, desolvation gas (nitrogen) 15 L/h, desolvation temperature 250°C, source temperature 200°C and interface voltage 4.5 kV. Selective ion recording using the combined signal from the most dominant peaks for a) DON [major peak Na^+^DON (m/z 319.2)] and for b) the IS, ^13^C_15_-DON [major peak Na^+13^C_15_-DON (m/z 334.2)] were used.

External standards of concentrations 0, 2, 5, 10, 20, 50, 100 and 250 ng/mL spiked with IS (8 ng/mL) were included at the start of each batch and a further IS-only sample was included at the end. Unknowns and QCs were adjusted for recovery using the IS. For all calibration curves, R^2^ was > 0.99. The limit of detection (LOD) was estimated at 0.5 ng DON/mL of urine.

## Results

### Urinary Biomarker Levels

The urinary biomarker levels are summarized in **Table 1**. Overall, the geometric mean (95% CI) of DON concentration was 2.03 (1.64 – 6.87) ng per mL urine. Ninety percent of the urine sample from this pilot study has detectable levels of urinary DON. Samples with urinary DON biomarker below LOD were assumed to have 0.25 ng/mL of DON (i.e. ½ LOD, LOD of the detection method: 0.5 – 62.5 ng/mL).

**Table 1.** Total urinary DON concentrations (ng/mL) and DON intake estimates (ng/kg bw/day) of the 30 university students

	**Urinary DON concentration**	**Estimated DON intake**
	(ng/mL)	(ng/kg bw/day)
**Geometric mean (95% CI)**	2.03 (1.64 – 6.87)	53.44 (59.24 – 256.62)
**Maximum**	28.49	1062.17
**Minimum**	0.25 (below LOD)	9.52 (< LOD)
	Positive rate 90%	% Exceeding PMTDI (1 µg/kg bw/day): 3.3%

### Free DON (fDON) and Total DON Ratio

The levels of fDON (i.e. unmetabolized DON) and total DON were analyzed in a subset of five urine samples and the ratio was calculated. Those samples were repeated for extraction without the enzyme digestion step in order to measure the fDON level. The mean ratio was 19% (range 14–25%). The fDON/DON levels in the subset of five urine samples with the highest DON concentrations were 3.3/19. The increasing trend in fDON is in agreement with the total DON increase. This part of the results is in agreement with the values reported elsewhere^6^^)^.

### Estimation of Dietary DON Intake

According to the literature^6^^)^, the DON intake is significantly correlated with urinary DON (***p*** < 0.005). Therefore, exposure assessment in relation to regulatory recommendations was subsequently conducted based on the assumption that 70%^5^^)^ of the dietary intake of DON was excreted in urine, the daily volume of urine was 1.6 L and the average body weight was 60 kg^17^^,^^18^^,^^19^^)^. The probable daily intake was calculated for each student based on the Eq. 1 and was compared with the PMTDI of 1000 ng/kg bw/day by JECFA.

$$\frac{\text{urinary DON}\left(\text{ng/mL}\right)\text{×volume of daily urine excretion (mL/day)}}{\text{body weight}\left(\text{kg bw}\right)\text{ × \% DON urine excretion rate}}$$ (Eq. 1)

**Fig. 1.**
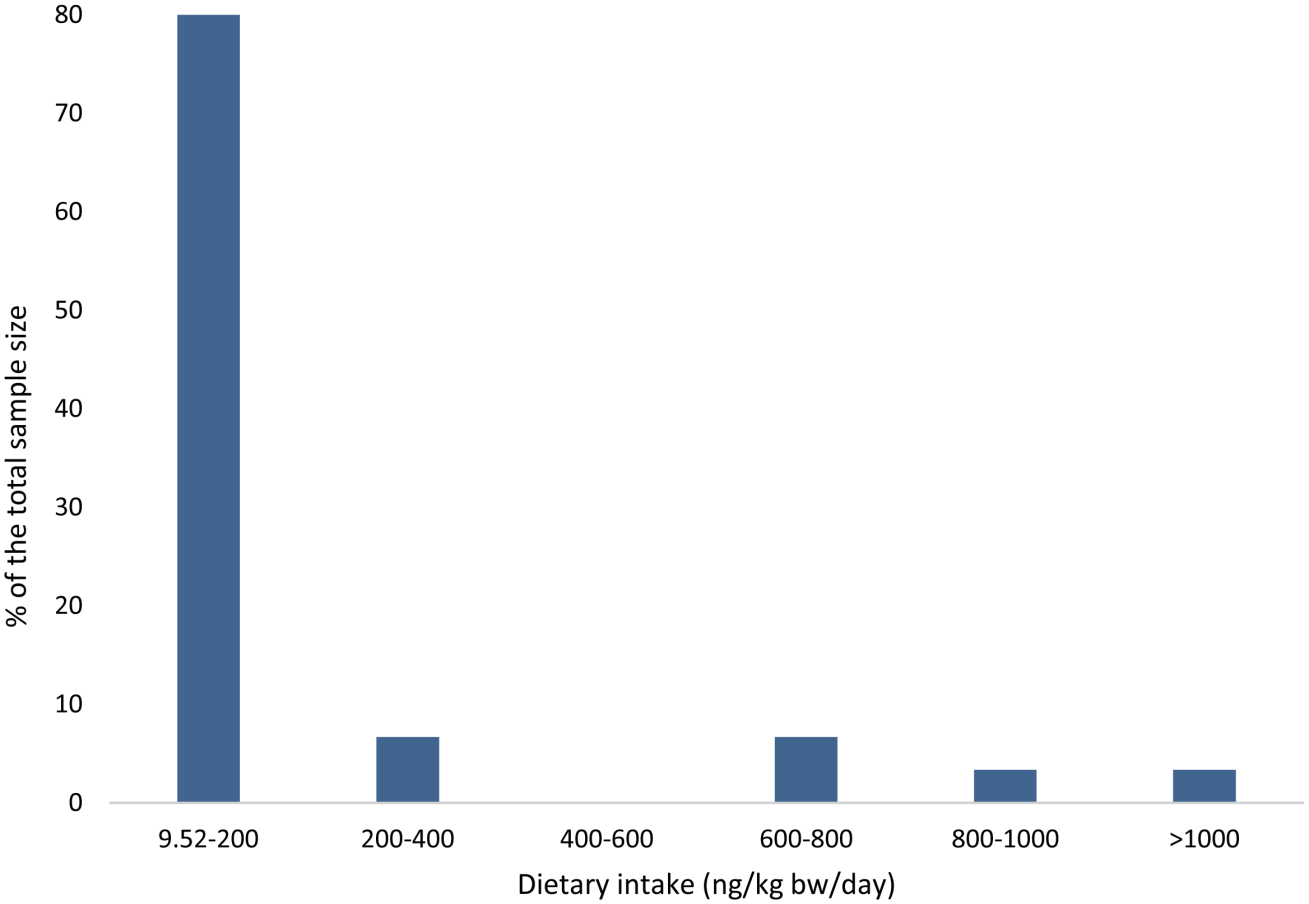
Percentage distribution of the 30 university students in different dietary DON exposure levels. Only one of the student showed the level higher than the PMTDI

Table 1 shows the urinary DON concentration and estimated dietary DON exposure of the 30 university students. Overall, the estimated geometric mean (95% CI) of DON intake is 53.44 (59.24 – 256.62) ng/kg bw/day. Only one of the sample suggests that the corresponding volunteer had a higher DON intake than PMTDI (1 µg/kg bw/day) which is 3.3% of the total sample size (The second highest exposure value is 818.55 ng/kg bw/day). Fig. 1 shows the distribution of the estimated DON intake. The majority of the population has a DON intake less than 200 ng/kg bw/day which is significantly lower than the PMTDI.

### Correlation between Urinary DON Level and Wheat Intake

**Fig. 2.**
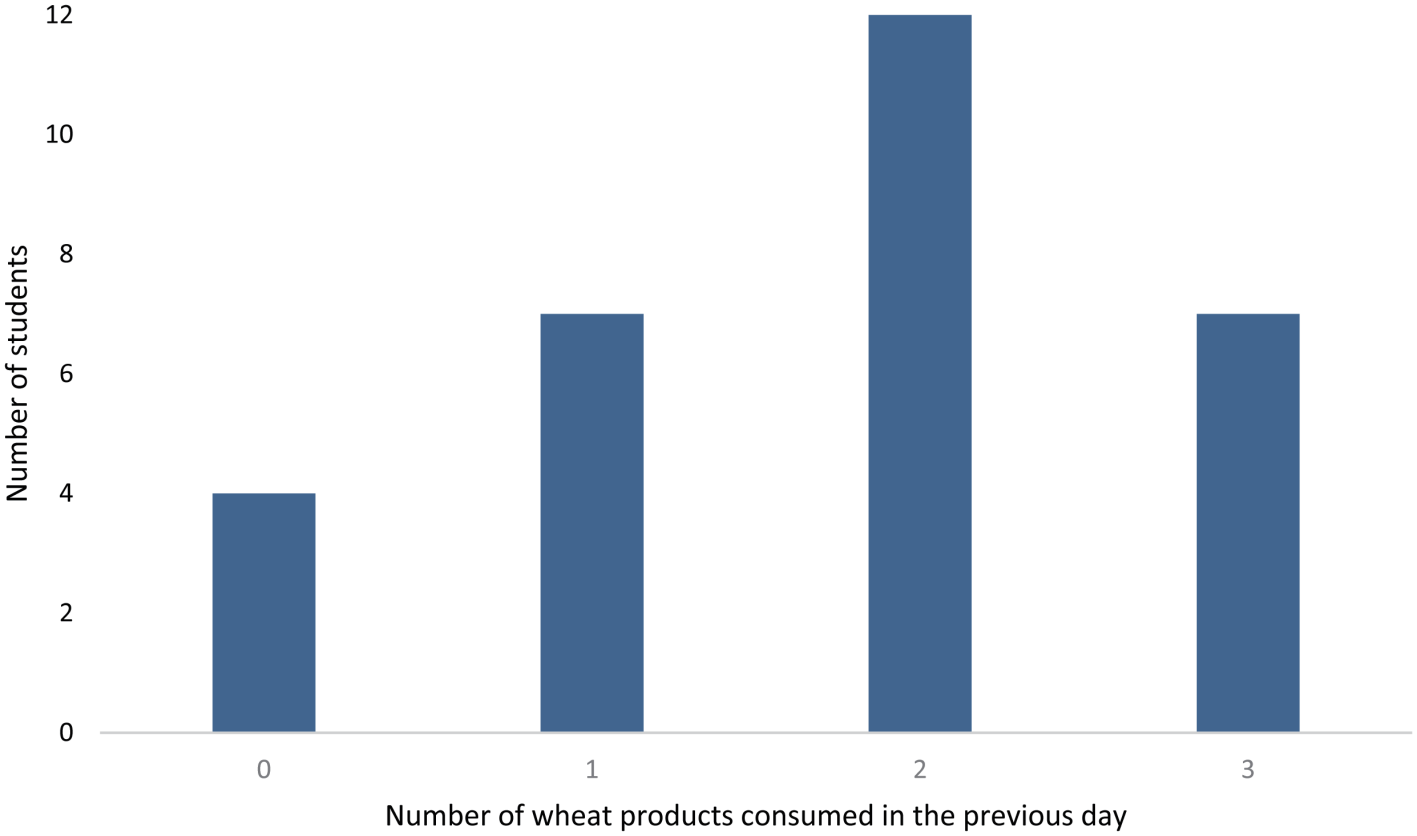
Number of wheat product consumed by each of the 30 university students on the day before urine sample collection, considering breakfast, lunch, afternoon tea and dinner

For the Japanese participants a possible correlation between last day wheat intakes and urinary DON levels were analyzed based on information provided in the dietary intake questionnaires. Due to the simple questionnaire provided, the last day wheat intake for each of the student was estimated as the wheat intake score: every wheat product consumed count as 1. The wheat intake score is the sum of the number of wheat product consumed during each meal (breakfast/lunch/afternoon tea/dinner), ranging from 0 to 3 for the students, as shown in **Fig. 2**. The wheat intake score was considered in Spearman correlation analysis together with the dietary DON exposure levels. Consumption of wheat products did not show a significant correlation with the urinary DON level of the subjects (***p*** = 0.94).

## Discussion

Urinary DON biomarkers have been shown to be a good indicator for DON exposure as they show a good correlation with food consumption especially with cereal, maize and wheat products^8^^,^^9^^,^^10^^,^^11^^,^^12^^,^^13^^,^^14^^,^^15^^)^. According to the dietary recall survey, the major source for DON exposure for the Japanese university students tested here was likely to be wheat products. However, no significant correlation was observed between the urinary DON level and wheat intake (***p*** = 0.94). It should be noted that only the number of wheat products consumed by the participant was recorded in the dietary recall survey and the sample size was small. In addition, other types of food products can also contribute to DON exposure and the level of DON or wheat composition can vary largely among foods that are marked as “wheat products”. Although most of the participants showed a very low level of DON in their urine, the estimated daily intake (EDI) for one sample with the highest urinary DON level still exceeded the PMTDI, which suggested that the DON exposure in the Japanese population may in some cases be high.

To put the detected urinary DON level in Japanese university students into wider context, the average urinary DON concentration is compared to other biomonitoring studies in Asia. Food contamination by *Fusarium* fungi has been found across the world. However, based on the difference in climatic factors (predominantly temperature and moisture) as well as dietary habits in different regions/countries, the distribution and prevalence of DON exposure are expected to vary among countries^20^^,^^21^^)^.

Among the biomonitoring studies conducted in Asia for urinary DON biomarkers, the levels reported from a study conducted in a “high risk” area (37 ng/mL) and a “low risk” area (12 ng/mL) in China^22^^)^ were clearly higher than those observed in this study. However, a more recent study which measured the biomarker level in Shanghai women showed a very similar result as this study (4.8 ng/mL^23^^)^). Another two studies conducted in Bangladesh both reported very low levels of urinary DON level in the participants (0.17 ng/mL^10^^)^; 0.86 ng/mL^12^^)^). The consumption of wheat and maize in the Bangladesh population is much lower than other countries, the only source of DON exposure in the Bangladesh population suggested by the authors to be from wheat and maize flours used to make bread^10^^)^.

In conclusion, the results of our study showed a relatively low level of DON exposure for the Japanese university students. The high positive rate of detectable urinary DON levels and the fact that one subject had the EDI of DON higher than the PMTDI still indicate that reducing the level of DON exposure is of importance from a public health perspective. Further studies are also required to investigate the possible health effect of chronic exposure to low levels of DON.
